# Color appearance shifts depending on surface roughness, illuminants, and physical colors

**DOI:** 10.1038/s41598-022-05409-2

**Published:** 2022-01-25

**Authors:** Youngjoo Chae

**Affiliations:** Department of Clothing & Textiles, Chungbuk National University, Cheongju, South Korea

**Keywords:** Materials science, Physics

## Abstract

Texture is an important synesthetic design element used in textile products. The three-dimensional surface of texture changes the amount and angle of reflected light causing a color appearance change from its original color. In this work, for a wide range of colors, it was quantitatively analyzed how the color appearances change depending on different textures and illumination, such as CIE standard illuminants A, F11, F2, and D65. It was found that strong-textured fabrics (with a surface roughness Ra of 0.46 mm) had larger hue appearance changes and consequent overall color appearance changes from their true colors due to illuminants than non-textured papers (with a surface roughness Ra of 0.03 mm). Between two types of fabrics with different textures of 0.21 and 0.46 mm, however, there was no significant difference in the magnitude of color appearance changes, indicating that the difference in surface roughness greater than 0.43 mm can produce significant differences in color appearance changes induced by illumination. It was also found that the magnitude and direction of color appearance changes under different CIE illuminants differed significantly according to the physical chroma and hue of the surface.

## Introduction

Science and technology have improved the speed and quality of the product manufacturing process, thereby expanding the scope of consumers’ product choices. As the industrial paradigm accordingly shifted to one that is consumer-centered, meeting the needs of various consumers through the development of preferable products and sales environment has been underway. In the fashion industry, in particular, designers have recently been making significant efforts to maximize the overall sensitivity of consumers by adding high levels of sensory features, including visual, tactile, aural, and olfactory features, to products. Particularly among the sensory features, the visual features of objects account for 80% of the information we perceive in our daily lives^1^ and draw purchasing decisions by primarily stimulating consumers in the environment where textile products are sold. Therefore, regarding the company, it is important to first enhance the visual features of the clothing product itself while creating an effective sales environment through product displays and lighting, whereby consumers can recognize the visual features more positively.

Among many visual features that affect the overall appearance of textiles, color is an essential design element that draws the commercial success of products^2^. Color is a concept that includes lightness, chroma, and hue and is an objectively quantifiable physical property and a subjective property that can be recognized differently under the influence of various factors such as size, shape, surface texture, background, and external lighting of the colored area^3–5^. Color as a subjective property is widely reported in vision and color science literature as "*color appearance*." Among the aforementioned factors affecting the color appearance of textiles, texture is a three-dimensional feature created by the yarns or fibers interlaced in various ways in the textile. The three-dimensional surface created by texture changes the amount and angle of reflected light when it meets illumination in the product sales environment, creating a color appearance different from a smooth surface without texture. We are familiar with the importance of illumination for color appearance; regardless of how identical the two products’ physical color attributes are, they do not appear identical under different illumination^6^. Some consumers may face confusion when the color of a clothing item seen at the store is not the same as that seen under different lighting at home. The importance of illumination is significant not only at the point of selling clothes, but also in the manufacturing process. Even if the designer and the manufacturer accurately perform color communication with designated color numbers, if the color communication is performed under different illumination, the manufacturer may create clothes in an unintended color, delaying the time of supply of the finished product and causing fabric waste. As such, we know that illumination causes color appearance changes in various environments, but we do not know precisely which illumination causes the color appearance to change, how much and in what direction, therefore a quantitative analysis of illumination effects is necessary. In addition, for useful utilization in the fashion industry, research is needed on the effect of illumination on color appearance changes considering the surface texture that determines tactile and aural qualities beyond the visual appearance of textile products.

There has not been abundant research in the textile field on the effect of illumination on color appearance changes. Jeong and Lee^7^ photographed seven differently colored fabrics under red, yellow, green, blue, and purple illuminants, and printed the photographs to measure the color of the fabrics therein. Results found that bright fabrics were more affected by colorful illuminants than dark fabrics, causing greater color changes. In the study by Choi et al.^8^, five differently-colored fabrics in red, yellow, green, blue, and purple were presented under white illuminants with three different correlated color temperatures (CCT). Subjects were instructed to rank the fabrics based on their similarity to the color appearance observed in natural light. The results found that, aside from red fabric, the fabrics were recognized to be more similar to the color observed in natural light under illuminants of higher CCT (where the color is bluer with higher CCT and more reddish with lower CCT). These two studies used fabrics of various colors to systematically analyze color inconstancy according to illumination by different methods. However, the two studies did not present the color appearances under illumination as standardized quantities, such as CIE (Commission Internationale de l'Elcairage) colorimetric values. Meanwhile, Chae^9^ presented the degree of color appearance changes of fabrics in 24 different hues, owing to illumination as standardized values. In addition, a method of predicting the color appearance values of fabrics under various illumination was proposed, and the accuracy of the method was verified. However, the aforementioned previous studies, including Chae^9^, did not consider the surface characteristics of fabrics. In another study by Chae^6^, the illumination effects on yarn color mixtures considering the surface characteristics of colored-yarn mixed fabrics were analyzed in comparison with solid-colored fabrics. However, this study intentionally used a smooth surface with a nearly invisible texture to exclude the effects of other factors and highlight the multi-colors of yarns appearing on fabric surfaces. As previously noted, texture is an important synesthetic design element used in textile products, and the interaction of texture and illumination must be considered.

This study quantitatively analyzed how the color appearances of textiles of different texture strengths (surface roughnesses) and a wide range of colors change under CIE standard illuminants, compared to non-textured papers. Specific objectives of this study are as follows. (1) Under different standard illuminants, examine whether lightness, chroma, and hue appearance changes occur at a degree higher than that at which they are detectable. (2) Analyze the difference between true physical color and color appearance under each standard illuminant. (3) Comparatively analyze the degrees of the color appearance changes caused by illumination, depending on the texture strength and physical lightness, chroma, and hue of textiles.

## Methodology

### Samples

Forty-eight fabric samples with two different texture strengths and 24 non-textured paper samples (Pantone Fashion, Home + Interiors Color Guide, FHIP110A), which were reference samples, were used. All the fabric samples were piece-dyed plain woven fabrics without luster, and their different texture strengths were mainly due to the different diameters of the yarns used, that is, 0.19 mm and 0.46 mm, and different yarn densities of 72 × 72 /inch and 16 × 13/inch. The reason for using plain weave (in which warp yarns, i.e., vertical yarns, cross weft yarns, i.e., horizontal yarns, by going over one, then under the next, and so on at right angles forming a simple criss-cross pattern) is that it is the simplest and the most common of all types of weave, and it can create regular surface textures in a variety of strengths by simply controlling the diameter and density of the yarns used. Additionally, lusterless samples (both fabric and paper samples) were preferred because luster reacts with illuminants and affects the overall physical colors and color appearances of the samples. Table 1 describes three types of differently textured samples, and Table 2 shows some examples of actual samples in 24 triplets (3 types × 24 color centers = 72 samples in total). The samples in each triplet were prepared to match to each other in terms of color using a Pantone CAPSURE color matcher (X-Rite, USA) (but it does not mean that the samples in each triplet have exactly the same physical color values; see Fig. 2 for the physical color attributes of 72 samples under illuminant D65).

**Table 1:** Description of three types samples.

Type	Number	Material	Thickness	Weave	Yarn diameter	Fabric density
Non-textured (paper samples)	24	Paper	0.21 mm	–	–	–
Weak-textured (fabric samples)	24	100% cotton	0.61 mm	Plain	0.19 mm	72 × 72 /inch
Strong-textured (fabric samples)	24	100% cotton	1.47 mm	Plain	0.46 mm	16 × 13/inch

**Table 2:**
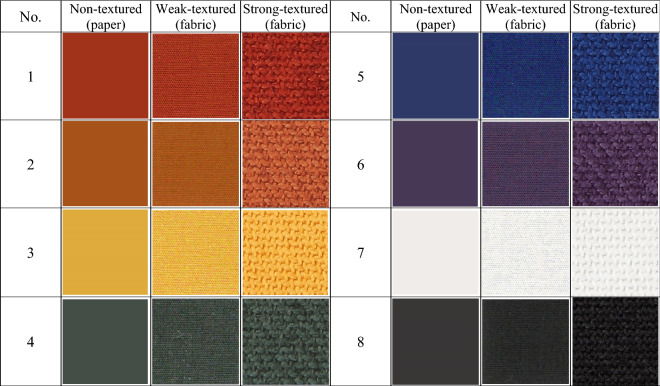
Examples of samples with different texture strengths.

Note that the scanned sample images might not faithfully represent the real colors measured in the experiment due to the different media on which the colors are represented.

### Quantification of texture strength

**Figure 1:**
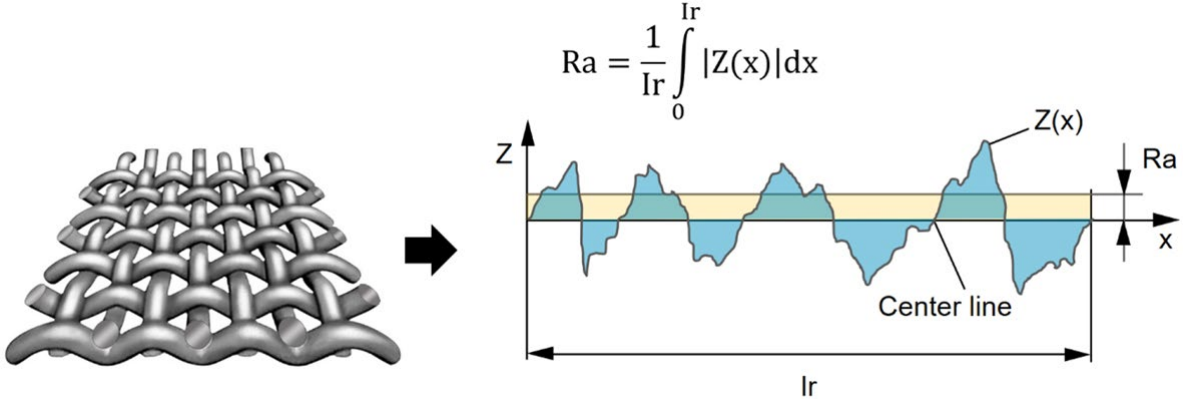
Derivation of the surface roughness parameter Ra of samples.

Three different texture strengths of samples were quantified by measuring the surface roughness with a Puotech 0918 surface roughness tester (China) based on the ISO 4287:1997 standard^10^. In the measurements, Ra, which is the arithmetic average of surface heights across the sample surface, was used as a roughness parameter. Figure 1 illustrates how the Ra of samples was derived from the height across the measured microscopic peaks and valleys. The derived roughnesses of non-textured, weak-textured, and strong-textured samples were 0.03 mm, 0.21 mm, and 0.46 mm, respectively, which correspond to the visually perceived roughnesses shown in Table 2.

### Physical color measurement

**Figure 2:**
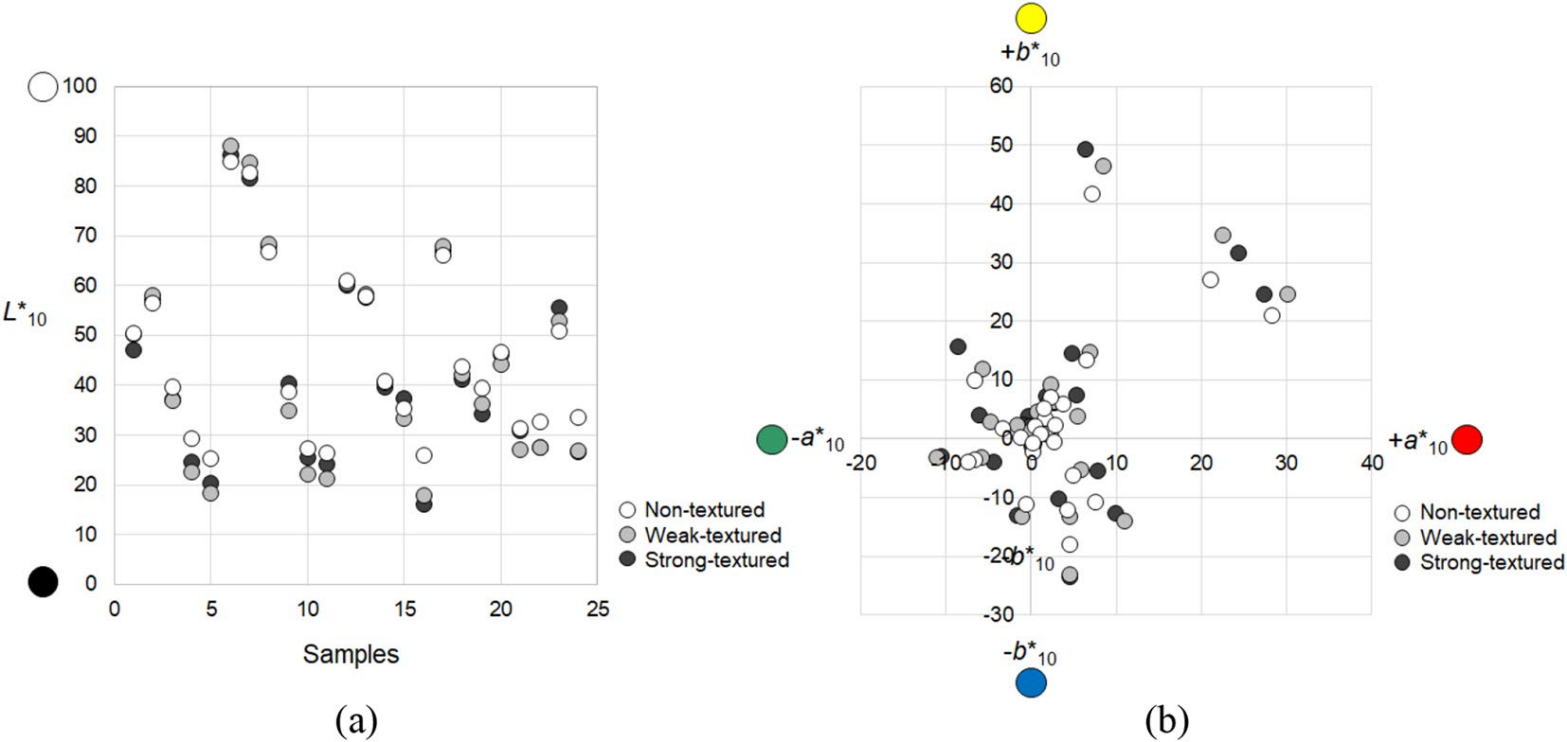
Distribution of 72 samples in the CIELAB color space under the illuminant D65: (**a**) L*_10_ and (**b**) a*_10_b*_10_ spaces.

The spectral reflectance values of 72 samples (48 fabric samples and 24 paper samples) were measured in a wavelength range from 360 to 740 nm with an interval of 10 nm by a Konica Minolta CM-26d spectrophotometer (Japan) with the following specifications: specular component included (SCI; this mode includes both the diffuse reflectance and specular reflectance during the color measurement process and is commonly used to obtain the true colors of colored stimuli); ultraviolet excluded; and a large aperture (MAV: 8 mm). From the reflectance values, the CIE lightness L*_10_, redness–greenness a*_10_, yellowness-blueness b*_10_, chroma C*_ab,10_, and hue *h*_ab,10_ values of samples were calculated based on the CIE 10° standard observer and the CIE standard illuminant D65. Figure 2 shows the 72 samples plotted in the CIELAB space according to their physical color attributes under the illuminant D65. D65 is commonly used in colorimetric applications to describe the true colors of colored objects and thus used in color communications in industry^3^. Meanwhile, all the measured reflectance data were used in the color appearance calculations of samples under other illuminants as discussed in the next section.

### Color appearance calculation

**Figure 3:**
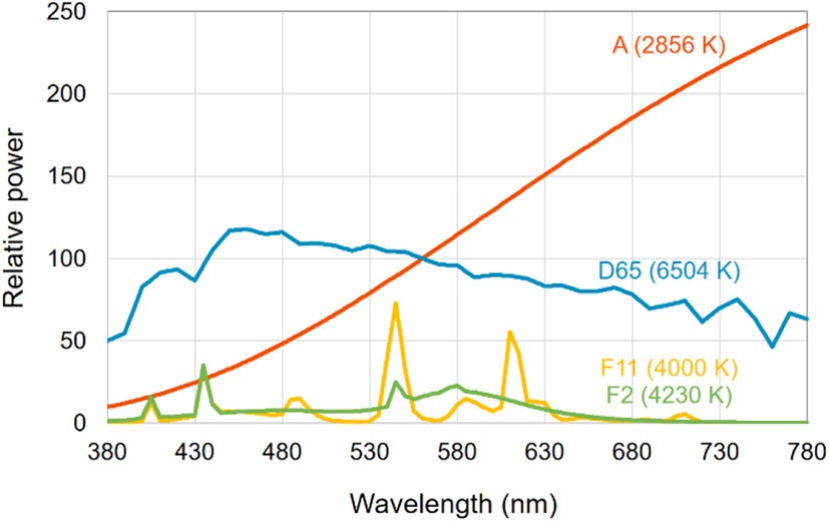
Relative spectral power distributions of the CIE standard illuminants used in the experiment: A (2856 K), F11 (= TL84; 4000 K), F2 (= CWF; 4230 K), and D65 (6504 K).

As color appearance attributes, the L*_10_, a*_10_, b*_10_, C*_ab,10_, and *h*_ab,10_ values of 72 samples under four different standard illuminants, that is, CIE illuminants A, F11, F2, and D65, were calculated based on the spectral data of the samples and illuminants, and the color matching functions^11^ of CIE 10° standard observer. The CIE illuminants A, F11, F2, and D65 are standardized representations of incandescent, triband fluorescent, cool-white fluorescent, and daylight sources and have correlated color temperatures of 2856 K, 4000 K, 4230 K, and 6504 K, respectively^3^. In particular, F11 and F2 are also known under the names TL84 and CWF and are typically found in fashion stores in Europe and the Americas, respectively. The relative spectral power distributions of the four types of illuminants^12^ are illustrated in Fig. 3.

**Figure 4:**
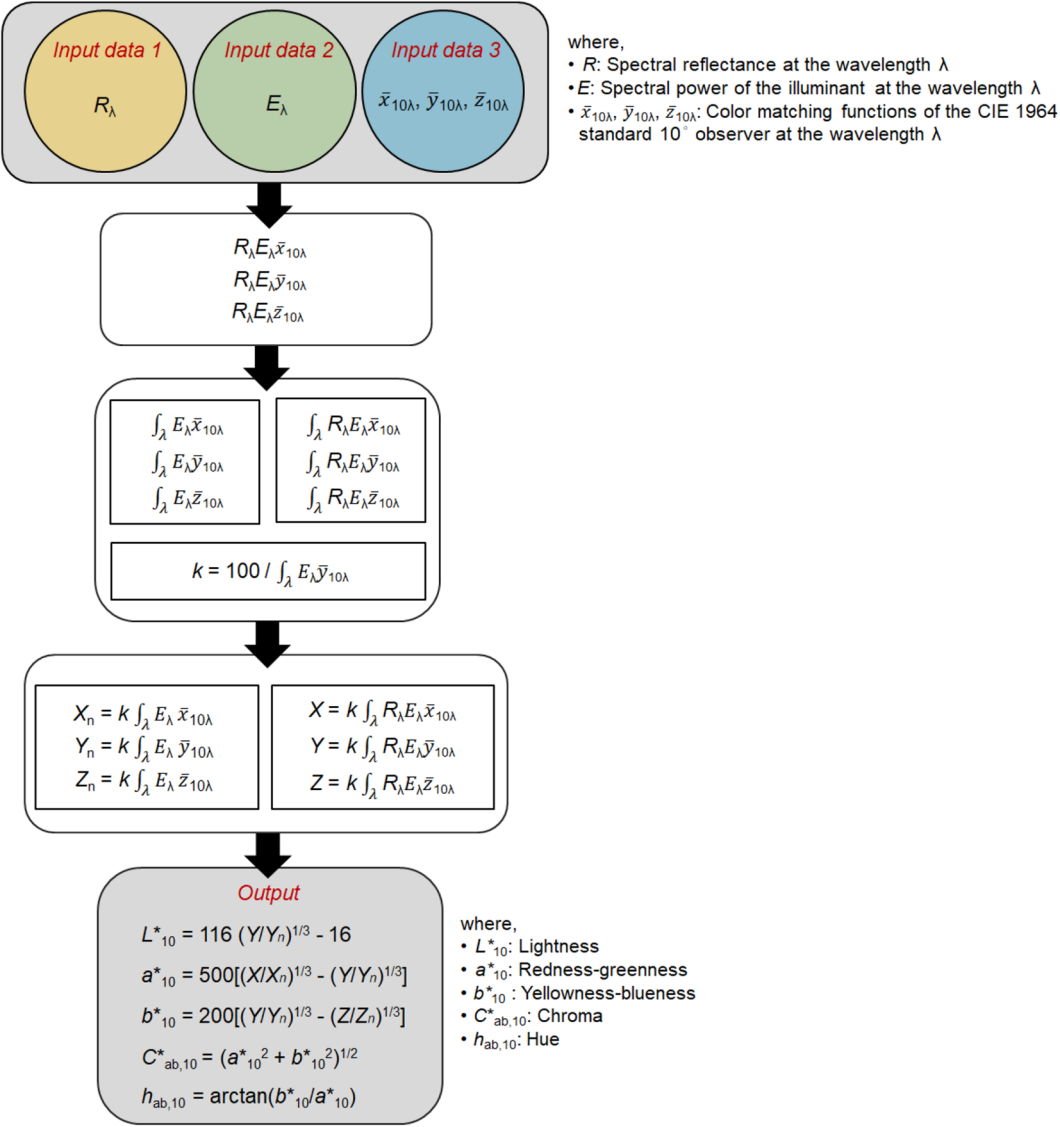
Flow chart of the calculation of L*_10_, a*_10_, b*_10_, C*_ab,10_, and *h*_ab,10_ values under a specific illuminant.

Meanwhile, Fig. 4 schematically illustrates how the CIE L*_10_, a*_10_, b*_10_, C*_ab,10_, and *h*_ab,10_ values of samples under each illuminant were calculated. In the calculations, the spectrophotometric data were used for samples (*Input data 1* in Fig. 4) and the CIE standard data^4,12^ were used for the spectral data of illuminants (*Input data 2*) and color matching functions of observers (*Input data 3*). In order to match the intervals with the spectral data regions of these three input items, data interpolation and extrapolation were performed.

### Data analysis

The magnitude of the color inconstancy of samples having different surface texture strengths under different standard illuminants, i.e., A, F11, F2, and D65, was numerically analyzed by subtracting the minimum value from the maximum value of each sample in each color attribute of lightness, chroma, and hue. Next, the overall color difference Δ*E*_CMC(2:1)_ between the spectrophotometrically measured colors, i.e., physical colors, and the colors calculated by considering the illumination, i.e., color appearances, of samples was calculated and then compared according to their texture strength. Then, statistical analyses were conducted by using IBM SPSS Statistics to examine the significant effects of illumination and sample factors on the color inconstancy of the samples. First, a Pearson’s correlation analysis was performed to determine the significant correlations between the variables. Then, a one-way analysis of variance (ANOVA) with Tukey's post-hoc test and a simple regression analysis were conducted to describe each of the significant correlations found.

## Results and discussion

### Magnitude of the color inconstancy of samples with changes in illumination

**Figure 5:**
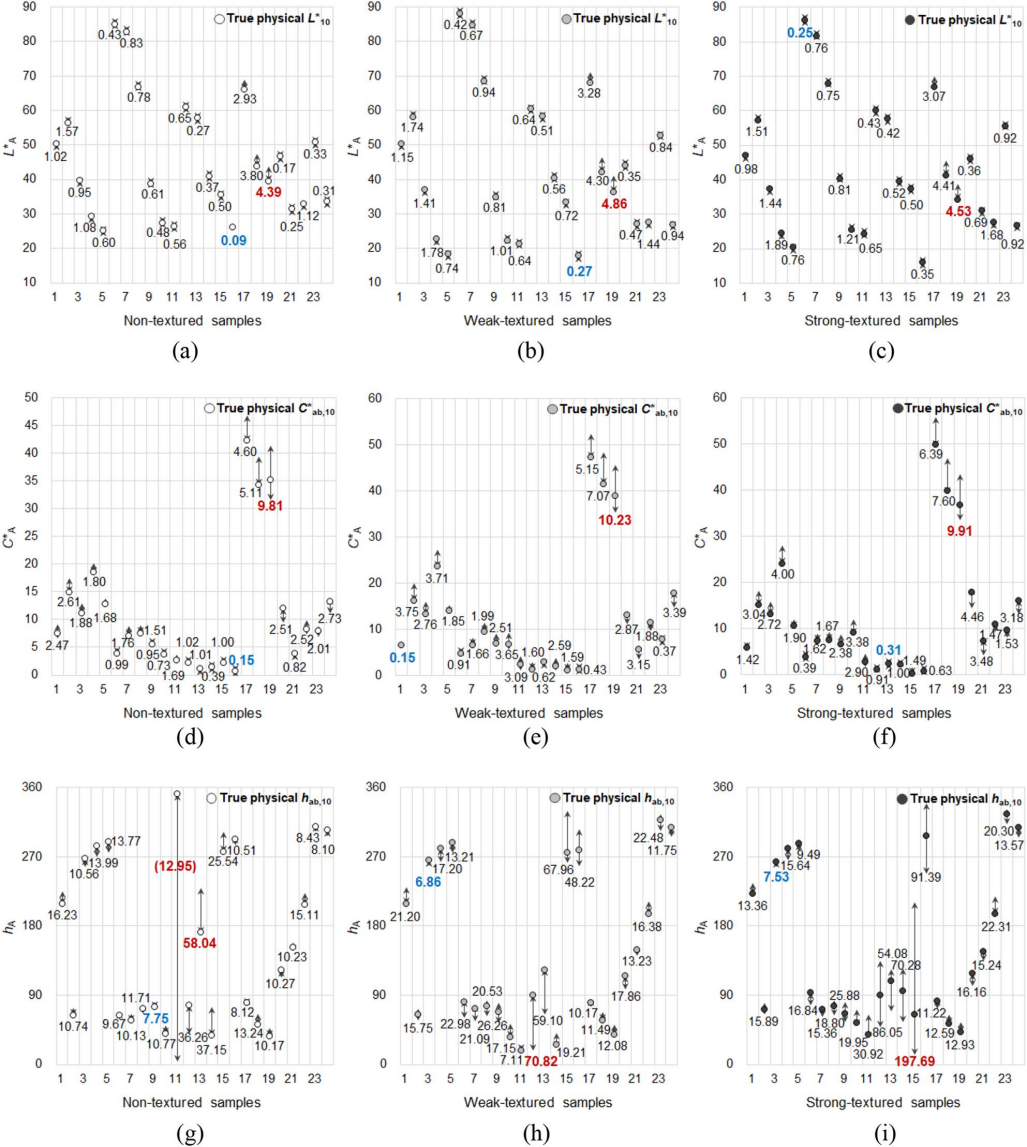
Ranges of the color appearances of samples with different surface texture strengths that vary under four different standard illuminants: (**a**) L*_A_ ranges of non-textured samples, (**b**) L*_A_ ranges of weak-textured samples, (**c**) L*_A_ ranges of strong-textured samples, (**d**) C*_A_ ranges of non-textured samples, (**e**) C*_A_ ranges of weak-textured samples, (**f**) C*_A_ ranges of strong-textured samples, (**g**) *h*_A_ ranges of non-textured samples, (**h**) *h*_A_ ranges of weak-textured samples, and (**i**) *h*_A_ ranges of strong-textured samples.

The color inconstancy of samples having different surface texture strengths was numerically analyzed by calculating the ranges of the varying lightness, chroma, and hue of the samples under four different CIE standard illuminants, that is, A, F11, F2, and D65. The color attributes that vary with the illuminant are different from physical color attributes, which are regarded as true colors, and embody perceptual color appearance attributes. To distinguish between these two concepts of constant physical color and inconstant color appearance attributes, the latter will henceforward be denoted by L*_A_, a*_A_, b*_A_, C*_A_, and *h*_A_. Meanwhile, the ranges of the varying L*_A_, C*_A_, and *h*_A_ of samples were calculated by subtracting the minimum value from the maximum value of each sample in the respective color appearance attributes. Figure 5 demonstrates the inconstant lightness, chroma, and hue induced by illuminants, in which L*_A_, C*_A_, and *h*_A_ changes ranged up to 4.86 (weak-textured fabric sample), 10.23 (weak-textured fabric sample), and 197.69 (strong-textured fabric sample), respectively. The wide scattering of L*_A_, C*_A_, and *h*_A_ shown in Fig. 5 indicates that the color appearances of samples were significantly affected by illuminants.

As can be seen in Fig. 5, each sample with different texture strengths and physical color attributes (see Fig. 2) had different L*_A_, C*_A_, and *h*_A_ ranges. The L*_A_ ranges of non-textured paper samples ranged from 0.09 to 4.39 (average: 1.00; S.D.: 1.12; see Fig. 5a), their C*_A_ ranges ranged from 0.15 to 9.81 (average: 2.16; S.D.: 2.01; see Fig. 5d), and their *h*_A_ ranges ranged from 7.75 to 58.04 (average: 15.81; S.D.: 11.98; see Fig. 5g). In the case of weak-textured fabric samples, the L*_A_ ranges ranged from 0.27 to 4.86 (average: 1.27; S.D.: 1.20; see Fig. 5b), the C*_A_ ranges ranged from 0.15 to 10.23 (average: 2.79; S.D.: 2.24; see Fig. 5e), and the *h*_A_ ranges ranged from 6.86 to 70.82 (average: 23.75; S.D.: 18.31; see Fig. 5h). Lastly, the L*_A_, C*_A_, and *h*_A_ ranges of strong-textured fabric samples were 0.25 to 4.53 (average: 1.24; S.D.: 1.17; see Fig. 5c), 0.31 to 9.91 (average: 2.82; S.D.: 2.34; see Fig. 5f), and 7.53 to 197.69 (average: 33.89; S.D.: 42.16; see Fig. 5i), respectively. Regardless of the texture strength of samples, when considering their maximum color appearance ranges, illuminants had huge effects on the color appearance changes of the samples. In particular, the maximum and even average ranges of chroma and hue appearances were far higher than the color discrimination thresholds of the human eye reported previously. According to the experimental results of Melgosa et al.^13^, chroma difference-thresholds ranged from approximately 0.7 to 1.2 ΔC*_ab,10_ depending on the hue, and smaller chroma differences were not perceived. Also, the hue difference-thresholds found by Montag and Berns^14^ and Qiao et al.^15^ ranged from approximately 1 to 3 Δ*h*_ab,10_. Thus, it can be assumed that people can easily detect the color appearance changes under different standard illuminants observed in this study.

Meanwhile, the 19th color center in all non-textured, weak-textured, and strong-textured sample sets, that is orange color (of which physical *h*_ab,10_ is close to 45; see Fig. 5g–i), had the maximum ranges of L*_A_ and C*_A_, indicating that the lightness and chroma appearances of the color varied most considerably depending on the illuminant. On the other hand, as for the hue appearance of samples, the color center with the largest hue appearance range depended on the texture strength of the sample—that is, the 13th, 12th, and 15th color centers for non-textured, weak-textured, and strong-textured sample sets, respectively. However, in the case of textured fabric samples, the color centers which had the largest hue appearance ranges, that is, the 12th and 15th color centers (of which physical *h*_ab,10_ were 89.99 and 64.41, respectively), were both close to yellow. All these results indicate that the magnitude of the illumination effect on the color appearance changes of samples varied depending not only on the texture strength of the samples, but also on their physical color attributes. It can also be seen in Fig. 5 that different samples with different texture strengths in different color centers had the different directions as well as the different magnitudes of color appearance changes from their true physical colors (indicated by circles). In other words, in each color appearance attribute, some samples had the single direction of color appearance changes, either positive or negative, while others had the both directions.

### Difference between the physical color and color appearance of samples under illumination

**Figure 6:**
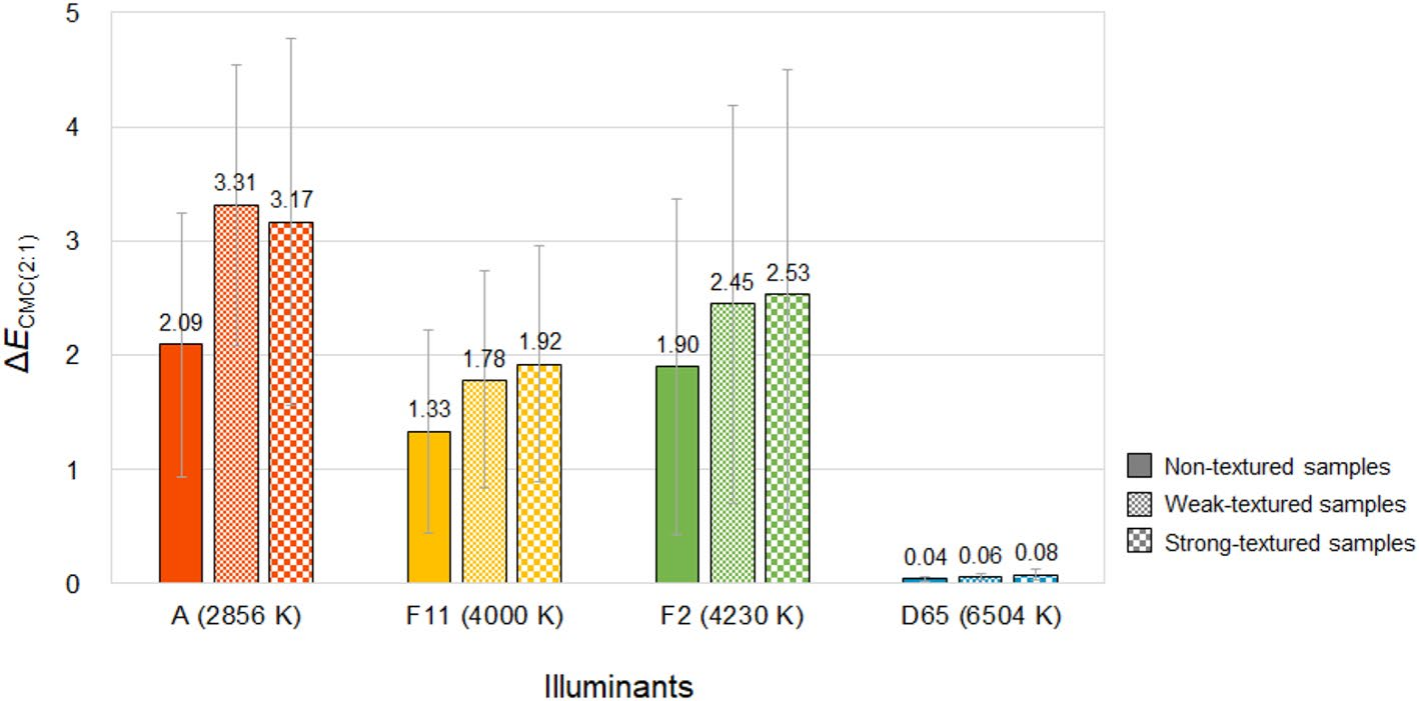
Overall color difference Δ*E*_CMC(2:1)_ between the physical colors and color appearances of samples with three different surface texture strengths (non-textured paper samples, weak-textured fabric samples, and strong-textured fabric samples) under four different standard illuminants (CIE illuminants A, F11, F2, and D65).

In order to show the discrepancy between true physical colors and color appearances induced by illuminants, the overall color differences Δ*E*_CMC(2:1)_ between the spectrophotometrically measured color values and the calculated color appearance values of 72 samples were calculated. Figure 6 compares the average Δ*E*_CMC(2:1)_ of samples with different texture strengths under different CIE standard illuminants. As can be seen in Fig. 6, different illuminants with different correlated color temperatures caused different magnitudes of the discrepancy. Regardless of the texture strength of samples, illuminant A with the lowest CCT of 2856 K caused the largest color appearance changes of samples from their true colors with the average Δ*E*_CMC(2:1)_ of 2.86. The smallest discrepancy under illuminant D65 of which the average Δ*E*_CMC(2:1)_ was almost zero is due to the fact that the spectrophotometric measurement of samples was conducted with the specification of CIE standard illuminant D65 to obtain their true colors. This small error also indicates that the method for calculating color appearance values used in this study is reliable and valid. Meanwhile, textured fabric samples had higher average Δ*E*_CMC(2:1)_ than that of non-textured paper samples, indicating that the magnitude of the effect of illuminants on the color appearance changes of samples differs according to whether the samples have a texture on the surface or not. Thus it is reasonable to say that the proper selection of illumination is particularly important in the sales environment of textured textile products.

### Illumination and sample factors affecting the color appearance changes of samples

To determine the statistically significant illumination and sample factors affecting the color appearance changes of the samples, a Pearson’s correlation analysis was performed. For independent variables, the correlated color temperature (CCT) of four CIE standard illuminants was considered as an illumination factor, and the texture strength (i.e., measured surface roughness) and the overall physical lightness L*_10_, chroma C*_ab,10_, and hue *h*_ab,10_ values of paper and fabric samples were considered as sample factors. For dependent variables, the lightness, chroma, hue, and overall color appearance changes of samples from their true physical colors induced by illuminants were used. Among the dependent variables, for lightness, chroma, and hue appearance changes, the absolute values, that is, $\left|{{\Delta L}^{*}}_{10}\right|$, $\left|{{\Delta C}^{*}}_{ab, 10}\right|$, and $\left|{\Delta h}_{ab, 10}\right|$, were used (overall color appearance change Δ*E*_CMC(2:1)_ always have positive values). That is because the illumination and sample factors have resulted in both positive and negative values of those color appearance changes (see Fig. 5), which would be deducted from each other when averaged. Table 3 shows Pearson’s correlation coefficients between the variables studied.

**Table 3:** Pearson’s correlation coefficients between the variables studied.

Independent variables		Dependent variables^b^			
		$\left|{{\Delta L}^{*}}_{10}\right|$	$\left|{{\Delta C}^{*}}_{ab, 10}\right|$	$\left|{\Delta h}_{ab, 10}\right|$	Δ*E*_CMC(2:1)_
Illumination factor	CCT	− 0.419**	− 0.480**	− 0.336**	− 0.627**
Sample factors^a^	Texture strength	0.021	0.056	0.160**	0.140*
	Physical L*_10_	− 0.010	0.003	− 0.053	− 0.087
	Physical C*_ab,10_	0.649**	0.577**	− 0.245**	0.472**
	Physical *h*_ab,10_	− 0.149*	− 0.151*	− 0.034	− 0.081

^a^Sample factors.

Notes. Texture strength: measured surface roughness.

Physical L*_10_, C*_ab,10_, and *h*_ab,10_: spectrophotometrically measured L*_10_, C*_ab,10_, and *h*_ab,10_ values of the sample.

^b^Dependent variables.

Notes. Color appearance changes of samples from their physical colors caused by illumination:

$$\left|{{\Delta L}^{*}}_{10}\right|$$ = $$\left|{{L}^{*}}_{\mathrm{A}}\mathrm{ under} \, \mathrm{ the} \, \mathrm{ illuminant }-\mathrm{ spectrophotometrically} \, \mathrm{ measured }{{L}^{*}}_{10}\right|$$; $$\left|{{\Delta C}^{*}}_{ab, 10}\right|$$=$$\left|{{C}^{*}}_{\mathrm{A}}\mathrm{ under} \, \mathrm{ the} \, \mathrm{ illuminant}-\mathrm{ spectrophotometrically} \, \mathrm{ measured }{{C}^{*}}_{ab,10}\right|$$;

$$\left|{\Delta h}_{ab, 10}\right| = \left|{h}_{\mathrm{A}}\mathrm{ under} \, \mathrm{ the} \, \mathrm{ illuminant}-\mathrm{ spectrophotometrically} \, \mathrm{ measured }{h}_{ab,10}\right|;$$

Δ*E*_CMC(2:1)_: Total color difference between physical color and color appearance.

*P < 0.05; **P < 0.01.

As presented in Table 3, the CCT of illuminants and the physical C*_ab,10_ of samples significantly affected the magnitudes of the changes in all the color appearance attributes of lightness, chroma, and hue appearances, and overall color appearance of samples at a significance level of 0.01 (*P* < 0.01). In particular, the effects of the CCT of illuminants found in this study were inconsistent with the findings of a previous study on the effect of illumination on the color appearance changes of fabrics ^6^. In the previous study, the effects of the CCT and luminance of illuminants were studied with the use of colored-yarn mixed woven fabrics and it was found that the CCT of illuminants affected only the lightness appearance changes of the fabrics. This indicates that different types of fabrics with different surface characteristics have different effects of illumination on their color appearance changes. Meanwhile, the texture strength of samples significantly affected their changes in hue and overall color appearances under illuminants, while the physical *h*_ab,10_ of samples significantly affected their lightness and chroma appearance changes (*P* < 0.05).

#### Effects of the correlated color temperature of illuminants

**Figure 7:**
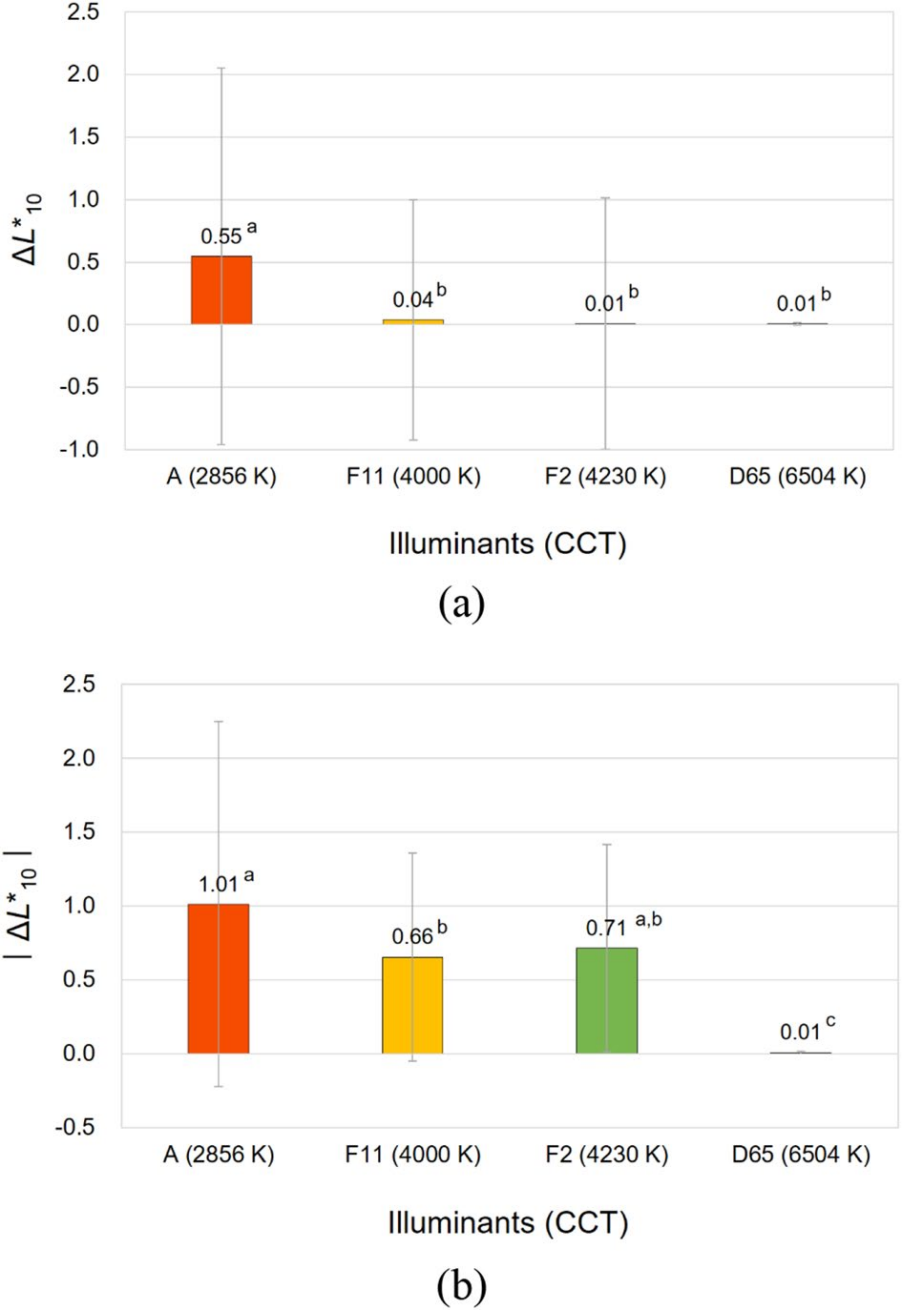
Effect of the correlated color temperature (CCT) of illuminants on the lightness appearance change, (**a**) ΔL*_10_ and (**b**) $\left|{{\Delta L}^{*}}_{10}\right|$, of samples. (ΔL*_10_ = L*_A_ under the illuminant—spectrophotometrically measured L*_10_).

**Figure 8:**
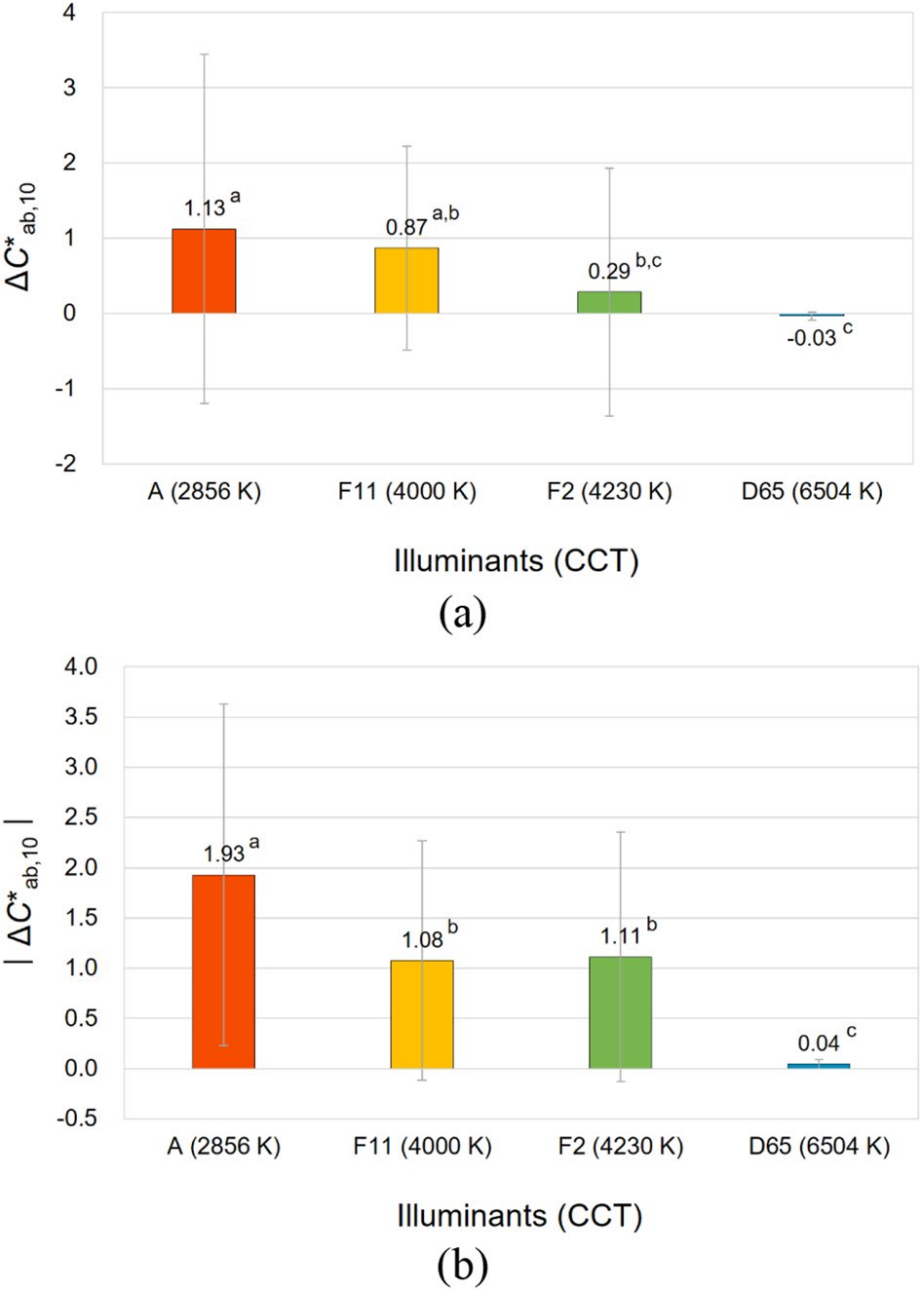
Effect of the correlated color temperature (CCT) of illuminants on the chroma appearance change, (**a**) ΔC*_ab,10_ and (**b**) $\left|{{\Delta C}^{*}}_{ab,10}\right|$, of samples. (ΔC*_ab,10_ = C*_A_ under the illuminant − spectrophotometrically measured C*_ab,10_).

**Figure 9:**
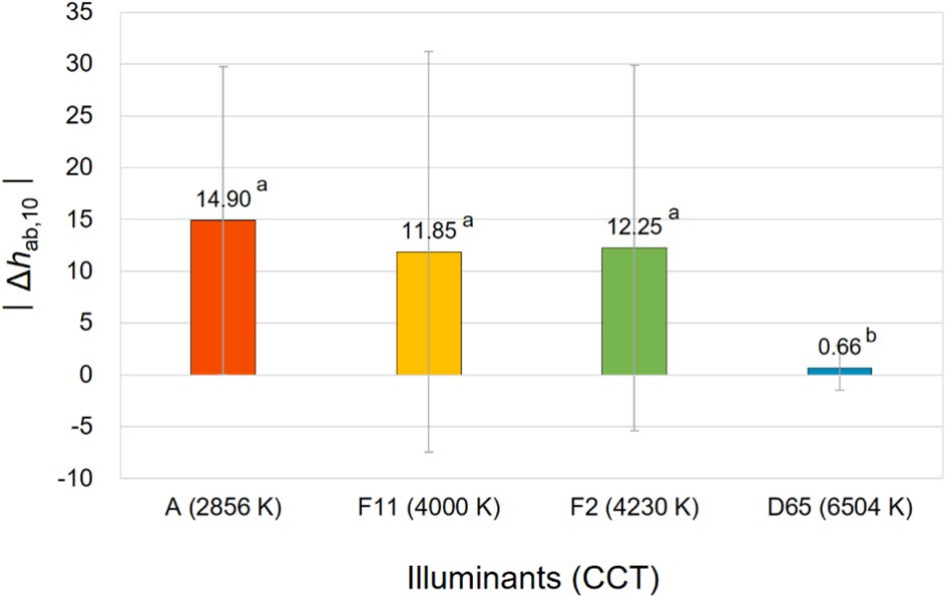
Effect of the correlated color temperature (CCT) of illuminants on the hue appearance change, $\left|{\Delta h}_{ab,10}\right|$, of samples. (Δ*h*_ab,10_ = *h*_A_ under the illuminant—spectrophotometrically measured *h*_ab,10_).

**Figure 10:**
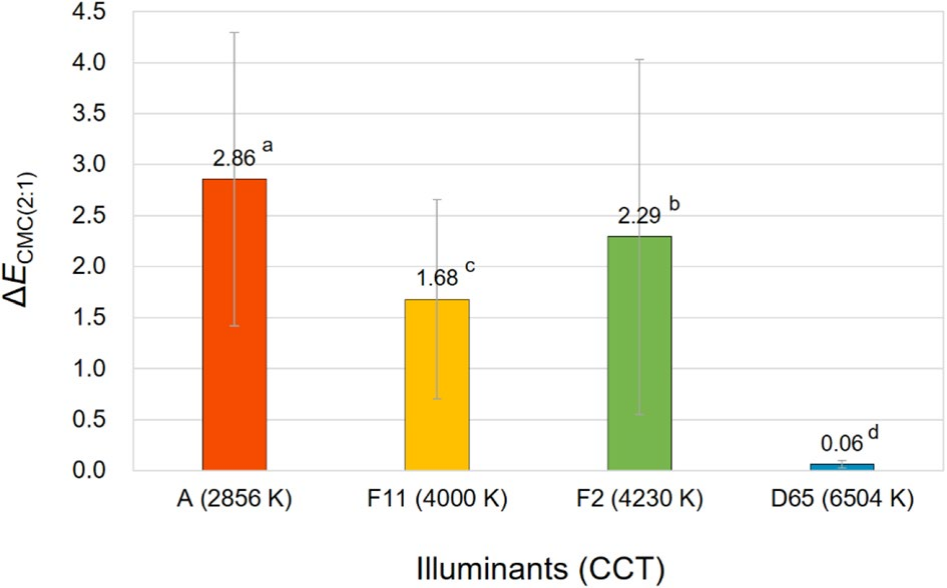
Effect of the correlated color temperature (CCT) of illuminants on the overall color appearance change, Δ*E*_CMC(2:1)_, of samples.

To illustrate the significant effects of the CCT of illuminants, a one-way ANOVA with Tukey's post-hoc test was conducted. Figures 7, 8, 9, and 10 compare the average magnitudes of color appearance changes, that is, lightness, chroma, hue, and overall color appearance changes, respectively, of samples from their true physical colors under 2856 K, 4000 K, 4230 K, and 6504 K illumination conditions. For the magnitudes of lightness and chroma appearance changes, the relative values of the changes, that is, ΔL*_10_ and ΔC*_ab,10_, as well as the absolute values, that is, $\left|{{\Delta L}^{*}}_{10}\right|$ and $\left|{{\Delta C}^{*}}_{ab, 10}\right|$, were analyzed to see not only the effect sizes, but also their different directions. On the other hand, for the magnitudes of hue appearance changes, the absolute values of the changes, that is, $\left|{\Delta h}_{ab, 10}\right|$, were observed. This is because *h*_ab,10_ indicates the degree of the four unique hues red (*h*_ab,10_ = 0 or 360), yellow (*h*_ab,10_ = 90), green (*h*_ab,10_ = 180), and blue (*h*_ab,10_ = 270), which are arranged orthogonally making four quadrants (red–yellow, yellow–green, green–blue, and blue–red). Therefore, unlike ΔL*_10_ and ΔC*_ab,10_, discussing whether the value of Δ*h*_ab,10_ is positive or negative is meaningless if the two hues to be compared belong to different quadrants^6^. Meanwhile, in Figures 7, 8, 9, and 10, the superscripts a, b, c, and d denote the four groups that were determined to be significantly different from each other by Tukey's test.

It can be seen in Figs. 7b and 8b that among four illuminants with different CCTs, illuminant A caused the largest magnitude of lightness and chroma appearance changes on average resulting in the largest overall color appearance change accordingly, which is shown in Fig. 10. This means that the lightness and chroma appearances of paper and fabric samples differed more greatly from their actual physical lightness and chroma under the reddish illuminant with the lowest CCT of 2856 K than other illuminants with higher CCTs, which have more bluish hues. This (the results presented in Figs. 7a and 8a as well) also implies that even if samples presented under illuminant A and other illumination conditions of higher CCTs have exactly the same physical color attributes, the sample under illuminant A tends to appear brighter and more colorful than others (since ΔL*_10_ and ΔC*_ab,10_ were calculated by subtracting the spectrophotometrically measured color values, that is, L*_10_ and C*_ab,10_, from the relevant color appearance values, that is, L*_A_ and C*_A_, respectively, under the illuminant). Meanwhile, it is of note that a lower CCT condition of illumination did not always result in larger color appearance changes from actual colors. As can be seen in Figs. 8b and 9, the average chroma and hue appearance changes induced by illuminants F11 and F2 with CCT of 4000 K and 4230 K, respectively, were not significantly different from each other. Furthermore, illuminant F2 with a higher CCT generally induced the larger overall color appearance change of samples than illuminant F11 as shown in Fig. 10. This inconsistent result is thought to be due to the minute difference between the CCTs of these two illuminants. Also, since each color appearance attribute is differently affected by the CCT of illuminants, it is safe to say that the desired lightness, chroma, and hue appearances of products can be obtained by changing the CCT of the illuminants differently in the sales environment. For example, increasing the CCT of the illuminant from 4000 to 6504 K, even though it is a relatively huge change, may not cause a significant change in the lightness appearance of products (see Fig. 7a), while it is likely to cause the significant chroma and hue appearance changes (see Figs. 8a and 9). The specific trend of the illumination effect according to the physical color attributes of samples will be discussed later.

#### Effects of the texture strength of samples

**Figure 11:**
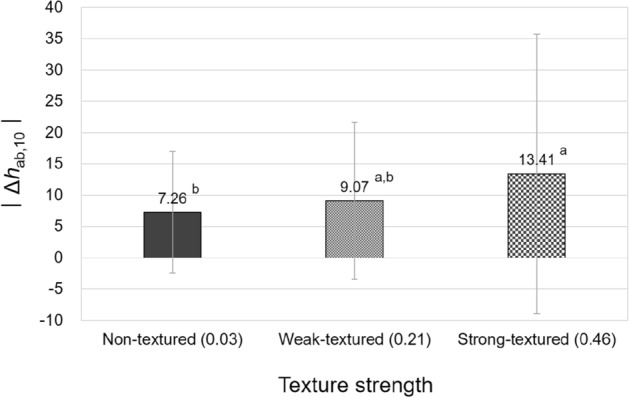
Effect of the texture strength of samples on their hue appearance change, $\left|{\Delta h}_{ab,10}\right|$, under illuminants. (Δ*h*_ab,10_ = *h*_A_ under the illuminant—spectrophotometrically measured *h*_ab,10_).

**Figure 12:**
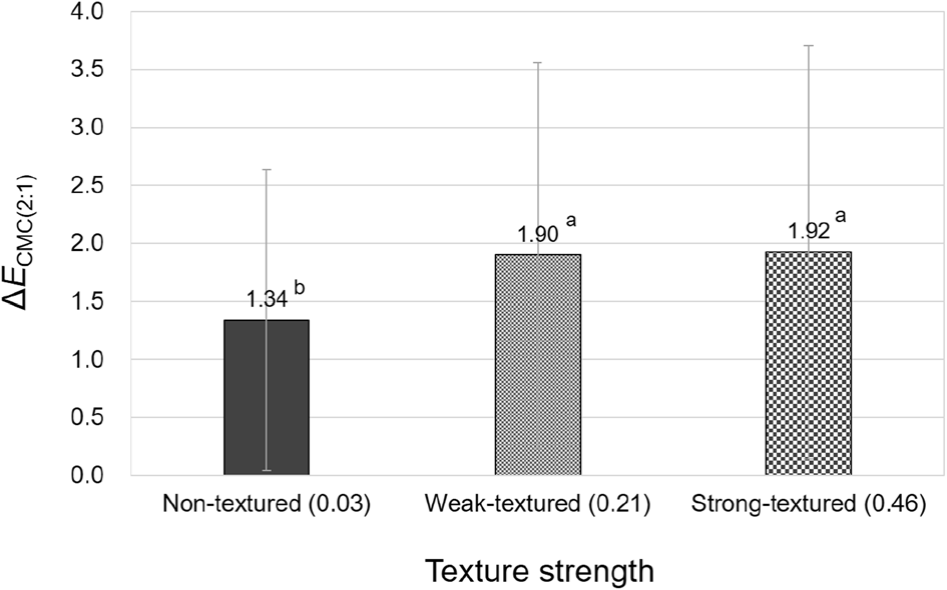
Effect of the texture strength of samples on their overall color appearance change, Δ*E*_CMC(2:1)_, under illuminants.

As stated previously, the texture strength of samples significantly affected their hue and overall color appearance changes under different illuminants. To illustrate these significant effects of texture strength, a one-way ANOVA with Tukey's post-hoc test was conducted. Figures 11 and 12 compare the average magnitudes of hue and overall color appearance changes, respectively, of three types of differently textured samples, non-textured paper samples and weak-textured and strong-textured fabric samples, under standard illuminants. The reason for using absolute values for hue appearance changes was discussed previously.

As can be seen in Figs. 11 and 12, strong-textured fabric samples had larger hue appearance changes and consequent overall color appearance changes from their true colors due to illuminants than non-textured paper samples. It is believed that the three-dimensional structure of the surface of strong-textured fabric samples, which is distinctly different from the two-dimensional surface of paper samples, reacted with illuminants and changed the direction and the amount of reflected light which determine the colors and color appearances of the surface. However, between the two types of textured fabric samples, namely weak-textured and strong-textured samples, there was no significant difference in the magnitude of color appearance changes. This indicates that when the difference in texture strength (Ra) of two surfaces is greater than 0.43 mm (strong-texture 0.46 mm—no-texture 0.03 mm = 0.43 mm), significant differences in the hue appearance and overall color appearance changes induced by illumination can be made. Meanwhile, it is interesting to note that there was no significant effect of texture strength on the lightness and chroma appearance changes under illuminants despite the fact that when three-dimensional textures of different heights meet with illumination, they create dark and desaturated shadows on the surface, which are likely to cause different degrees of overall lightness and chroma appearance changes of the surface. This is thought to be due to the not tremendously large difference in texture strength between samples, and further investigation with the use of more diverse samples in terms of texture strength will be useful to obtain more reliable results.

#### Effects of the physical C*_ab,10_ of samples

**Figure 13:**
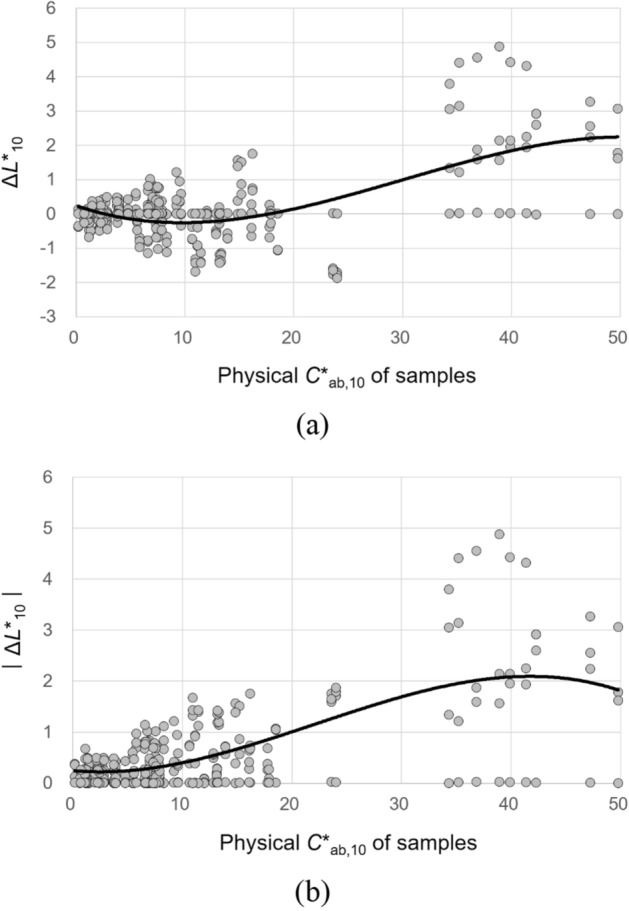
Effect of the physical C*_ab,10_ of samples on their lightness appearance change, (**a**) ΔL*_10_ and (**b**) $\left|{{\Delta L}^{*}}_{10}\right|$, under illuminants. (ΔL*_10_ = L*_A_ under the illuminant—spectrophotometrically measured L*_10_).

**Figure 14:**
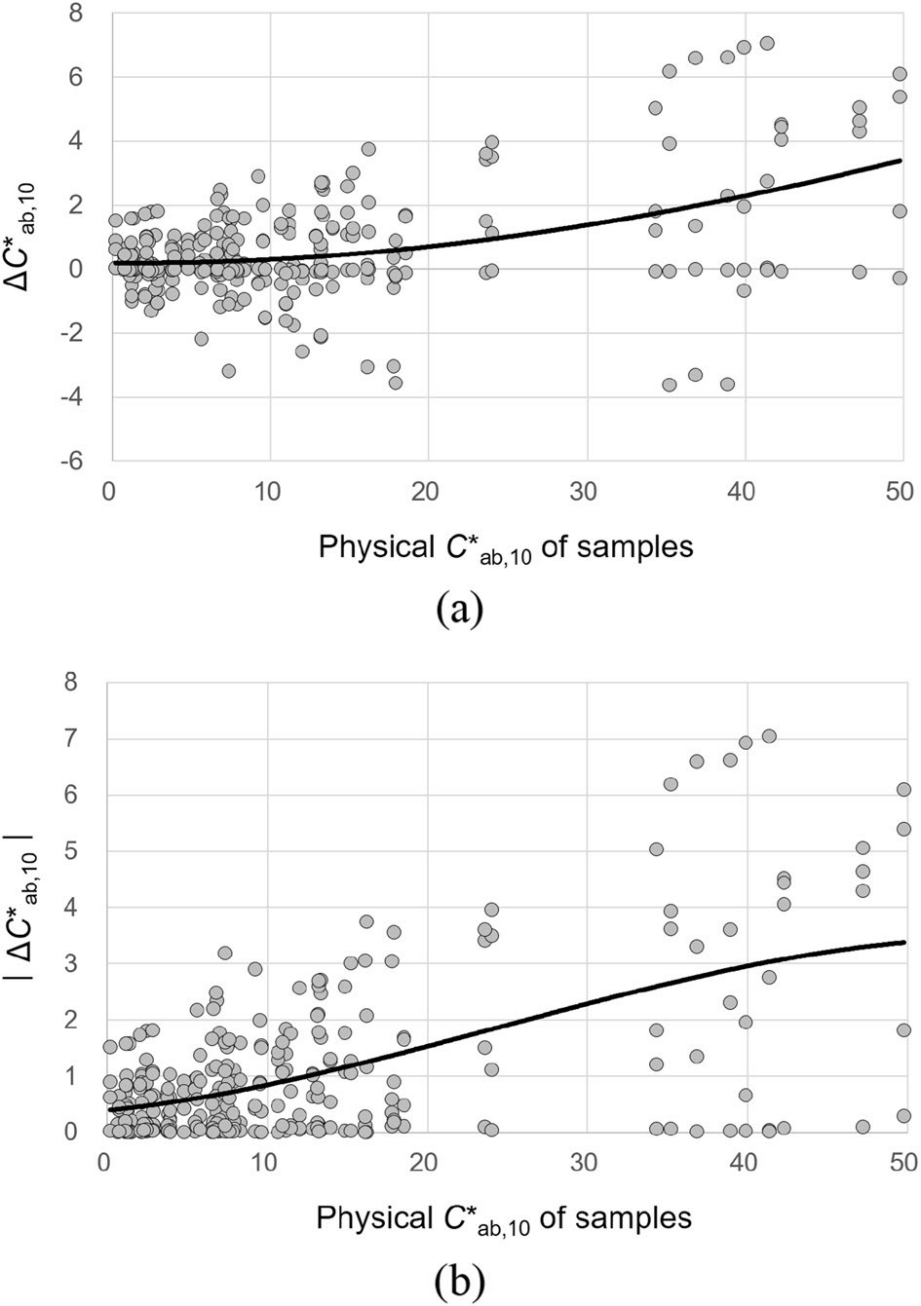
Effect of the physical C*_ab,10_ of samples on their chroma appearance change, (**a**) ΔC*_ab,10_ and (**b**) $\left|{{\Delta C}^{*}}_{ab,10}\right|$, under illuminants. (ΔC*_ab,10_ = C*_A_ under the illuminant − spectrophotometrically measured C*_ab,10_).

**Figure 15:**
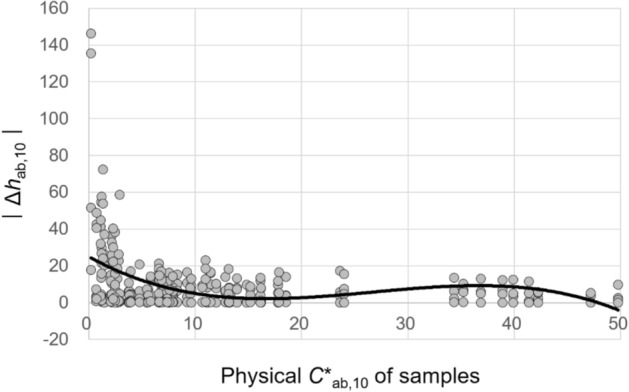
Effect of the physical C*_ab,10_ of samples on their hue appearance change, $\left|{\Delta h}_{ab,10}\right|$, under illuminants. (Δ*h*_ab,10_ = *h*_A_ under the illuminant − spectrophotometrically measured *h*_ab,10_).

**Figure 16:**
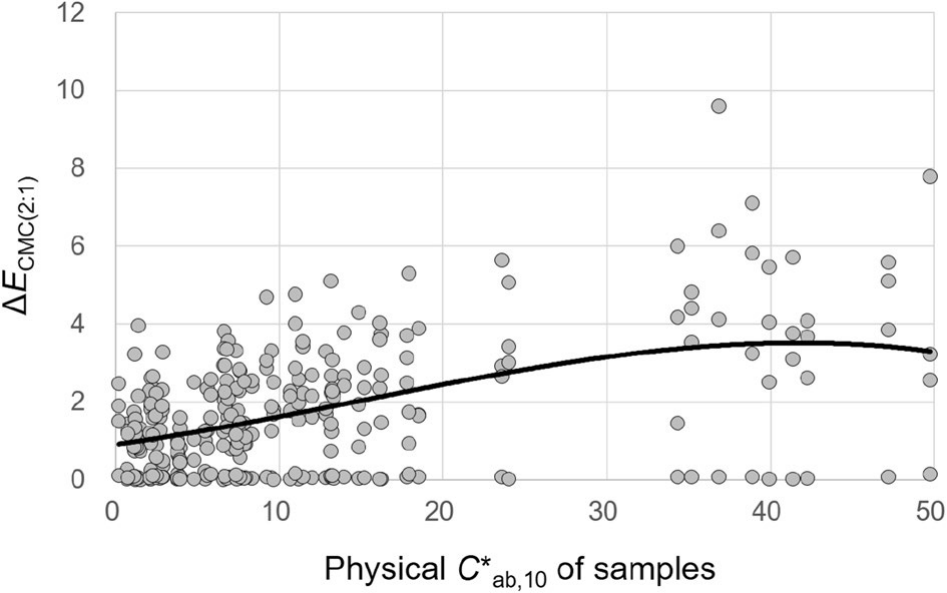
Effect of the physical C*_ab,10_ of samples on their overall color appearance change, Δ*E*_CMC(2:1)_, under illuminants.

**Figure 17:**
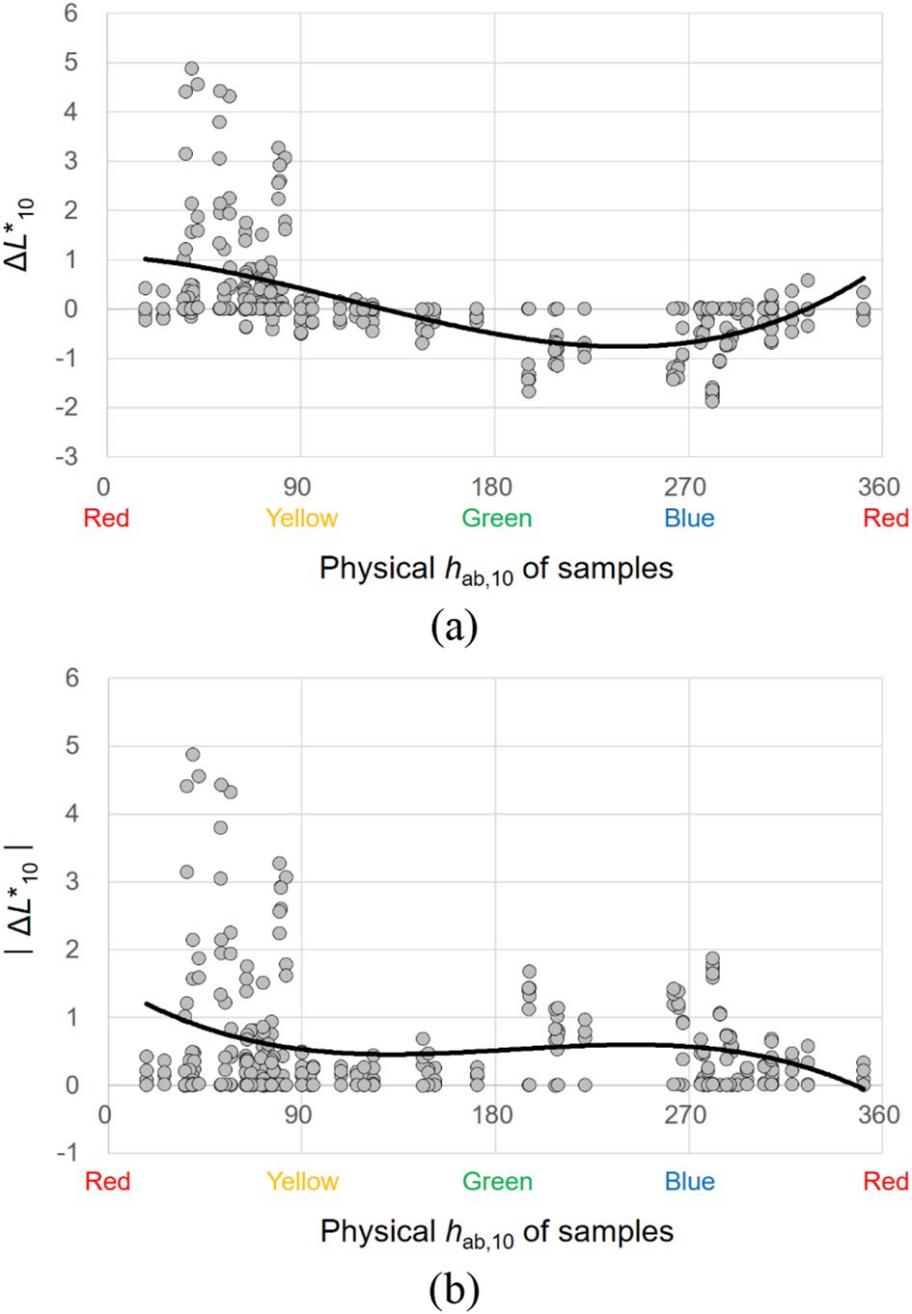
Effect of the physical *h*_ab,10_ of samples on their lightness appearance change, (**a**) ΔL*_10_ and (**b**) $\left|{{\Delta L}^{*}}_{10}\right|$, under illuminants. (ΔL*_10_ = L*_A_ under the illuminant − spectrophotometrically measured L*_10_).

**Figure 18:**
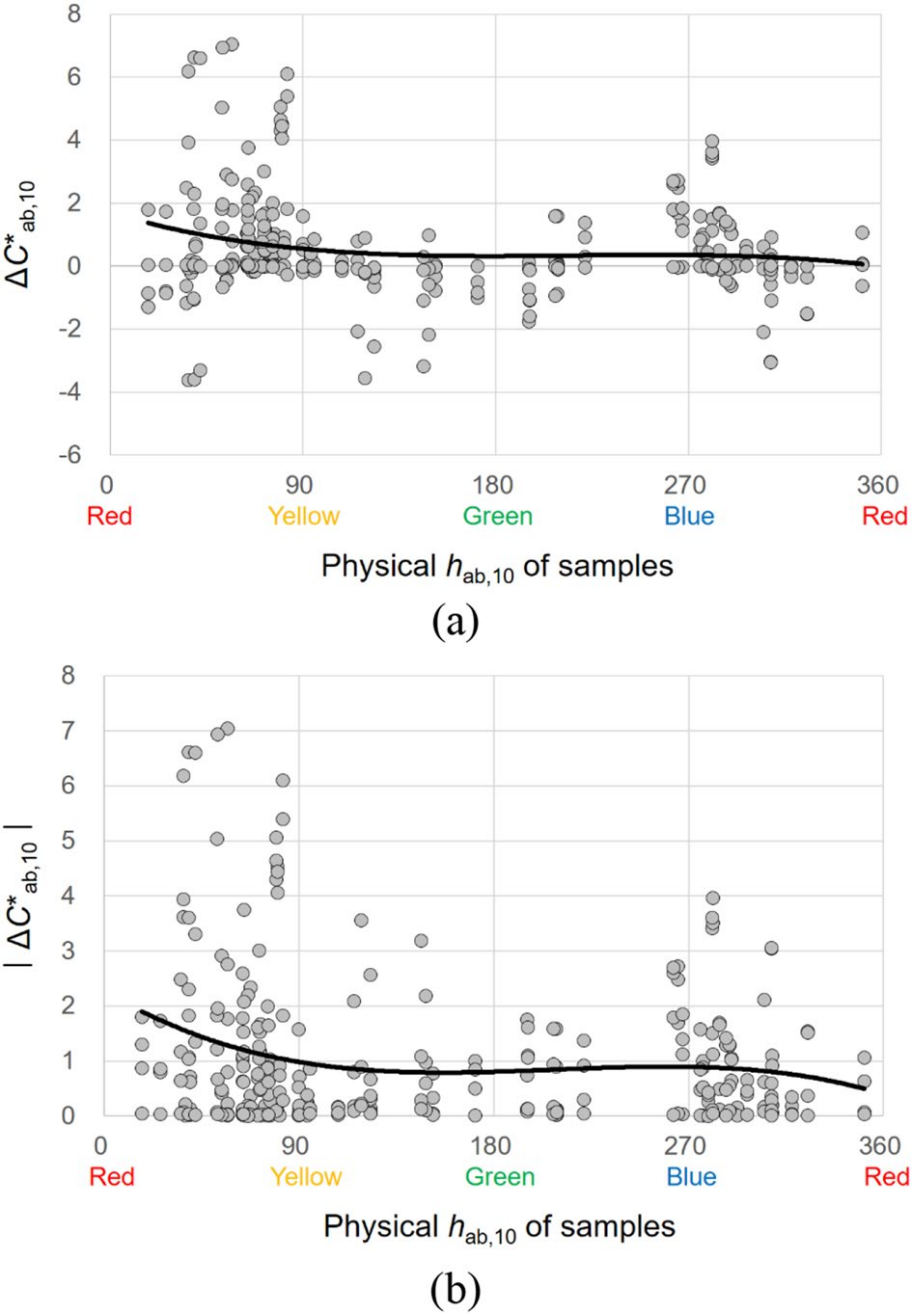
Effect of the physical *h*_ab,10_ of samples on their chroma appearance change, (**a**) ΔC*_ab,10_ and (**b**) $\left|{{\Delta C}^{*}}_{ab,10}\right|$, under illuminants. (ΔC*_ab,10_ = C*_A_ under the illuminant − spectrophotometrically measured C*_ab,10_).

The physical chroma C*_ab,10_ of samples significantly affected the magnitudes of all their lightness, chroma, hue, and overall color appearance changes under different CIE illuminants. To describe these effects, a simple regression analysis was conducted. Figures 13, 14, 15, and 16 show the plots of ΔL*_10_, ΔC*_ab,10_, Δ*h*_ab,10_, and Δ*E*_CMC(2:1)_, respectively, of 72 samples induced by illumination against the C*_ab,10_ of the samples. The least square method was employed to derive the best trend line for describing each of the significant effects. The first-, second-, and third-order polynomial functions were tried, and the third-order one given in Figures 13, 14, 15, and 16 (also in Figs. 17 and 18) was found to be the most reasonable to fit the sets of data points by minimizing the sum of squares.

The trend lines in Figs. 13b and 14b indicate that the magnitude of the lightness and chroma appearance changes of samples induced by illumination generally increased with the increment of C*_ab,10_ of the samples. As for the direction of these effects, Figs. 13a and 14a show that, under the CIE illuminants, samples had lighter and more saturated appearances than the actual colors having positive values of ΔL*_10_ and ΔC*_ab,10_ in general, and samples with higher physical chroma showed this trend more strongly. These greater lightness and chroma appearance changes of the samples of higher C*_ab,10_ resulted in greater overall color appearance changes as shown in Fig. 16. Meanwhile, the hue appearance changes of samples induced by illumination showed a different trend from their lightness, chroma, and overall color appearance changes—that is, samples with higher C*_ab,10_ did not always had larger hue appearance changes. It was also found that when the C*_ab,10_ of samples was close to 0, which indicates achromatic colors, the hue appearances of the samples were most affected by illumination overall having the widest $\left|{\Delta h}_{ab,10}\right|$ distribution and its highest average value of approximately 25. The inconstancy of achromatic colors in terms of hue appearances due to illumination was also reported by Chae and Lee^16^. On the other hand, when the C*_ab,10_ of samples was between 10 and 20 and close to 50, their hue appearances were least affected by illumination, maintaining their original physical hues.

#### Effects of the physical *h*_ab,10_ of samples

The physical hue *h*_ab,10_ of samples significantly affected the magnitudes of their lightness and chroma appearance changes under different CIE illuminants. Figures 17 and 18 show the plots of ΔL*_10_ and ΔC*_ab,10_, respectively, of 72 samples against the *h*_ab,10_ of the samples with the best fitting lines. As can be seen in Figs. 17b and 18b, when the *h*_ab,10_ of samples was between 0 and 90, which indicates orangish hues, the lightness and chroma appearances of the samples were generally the most different from their actual colors under the illuminants. On the other hand, when samples were of bluish-red hues having the *h*_ab,10_ between 270 and 360, relatively small lightness and chroma appearance changes were caused. Meanwhile, as for the direction of lightness appearance changes under the CIE illuminants, Fig. 17a shows that samples with the *h*_ab,10_ between approximately 0 (red) and 135 (yellowish-green) and between approximately 315 (bluish-red) and 360 (red) tended to have lighter appearances than their actual colors having positive values of ΔL*_10_. The samples of which *h*_ab,10_ was between approximately 135 (yellowish-green) and 315 (bluish-red), however, generally had darker appearances than the actual colors. Unlike the direction of lightness appearance changes, that of chroma appearance changes, which is described in Fig. 18a, indicates that samples generally had more chromatic appearances than their actual colors under the CIE illuminants regardless of their physical hue. All the results found in this study imply different effects of illumination on color appearances depending on the surface texture and physical color attributes and thus illumination can be used effectively so that objects with different surface characteristics can have intended color appearances.

## Conclusion

The diverse appearances of fabrics of different texture strengths and a wide range of colors under varied standard illuminants were quantitatively analyzed and compared to those of non-textured papers. As the illuminants, CIE illuminants A, F11, F2, and D65 with correlated color temperatures of 2856 K, 4000 K, 4230 K, and 6504 K, respectively, which cover from a reddish light to a bluish light, were employed. It was observed that each type of samples with different texture strengths and physical color attributes had different trends of color appearance changes due to illuminants. Generally, strong-textured fabrics with a surface roughness Ra of 0.46 mm had larger hue appearance changes and consequent overall color appearance changes from their true colors than non-textured papers with a Ra of 0.03 mm. Between two types of fabrics with different textures of 0.21 and 0.46 mm, however, there was no significant difference in the magnitude of color appearance changes, indicating that the difference in surface roughness greater than 0.43 mm can produce significant differences in color appearance changes induced by illumination. It was also found that the magnitude and direction of color appearance changes under different CIE illuminants differed significantly according to the physical chroma and hue of the surface. For example, samples in orangish hues with high physical chroma generally had large lightness and chroma appearance changes from their true colors.

The magnitudes and directions of illumination effects found in this work will allow the prediction of the variable color appearances of surfaces with different textures and physical color attributes under standard illuminants. This is envisaged to be advantageous to provide consumers with preferable products of intended color appearances by controlling illumination differently according to the surface texture of the products in the sales environment. However, since only limited types of texture were studied, further investigation on illumination effects with the use of a wider range of textures and other surface characteristics will be useful to produce more reliable results.

## Data Availability

The author confirms that the data supporting the findings of this study are available within the article.
